# The *SOD1*-mediated ALS phenotype shows a decoupling between age of symptom onset and disease duration

**DOI:** 10.1038/s41467-022-34620-y

**Published:** 2022-11-12

**Authors:** Sarah Opie-Martin, Alfredo Iacoangeli, Simon D. Topp, Olubunmi Abel, Keith Mayl, Puja R. Mehta, Aleksey Shatunov, Isabella Fogh, Harry Bowles, Naomi Limbachiya, Thomas P. Spargo, Ahmad Al-Khleifat, Kelly L. Williams, Jennifer Jockel-Balsarotti, Taha Bali, Wade Self, Lyndal Henden, Garth A. Nicholson, Nicola Ticozzi, Diane McKenna-Yasek, Lu Tang, Pamela J. Shaw, Adriano Chio, Albert Ludolph, Jochen H. Weishaupt, John E. Landers, Jonathan D. Glass, Jesus S. Mora, Wim Robberecht, Philip Van Damme, Russell McLaughlin, Orla Hardiman, Leonard van den Berg, Jan H. Veldink, Phillippe Corcia, Zorica Stevic, Nailah Siddique, Vincenzo Silani, Ian P. Blair, Dong-sheng Fan, Florence Esselin, Elisa de la Cruz, William Camu, Nazli A. Basak, Teepu Siddique, Timothy Miller, Robert H. Brown, Ammar Al-Chalabi, Christopher E. Shaw

**Affiliations:** 1 Department of Basic and Clinical Neuroscience, Maurice Wohl Clinical Neuroscience Institute, Institute of Psychiatry, Psychology and Neuroscience, King’s College London, London, SE5 9NU UK; 2 Department of Biostatistics and Health Informatics, Institute of Psychiatry Psychology & Neuroscience, King’s College London, SE5 8AF London, UK; 3 NIHR Biomedical Research Centre at South London and Maudsley NHS Foundation Trust and King’s College London, London, UK; 4 Homerton University Hospital, Homerton Row, London, E9 6SR UK; 5 Department of Molecular and Clinical Pharmacology, University of Liverpool, Blue Block 1.09, Sherrington Building, Crown St, Liverpool, L693BX UK; 6 Institute of Medicine, North-Eastern Federal University, 58 Belinsky str, Yakutsk, 677000 Russia; 7 Macquarie University Centre for MND Research, Macquarie Medical School, Faculty of Medicine, Health and Human Sciences, Macquarie University, Sydney, NSW Australia; 8 Department of Neurology, Washington University School of Medicine, St Louis, MO 63110 USA; 9 Concord Clinical School, ANZAC Research Institute, Concord Repatriation Hospital, Sydney, NSW 2139 Australia; 10 Department of Neurology and Laboratory of Neuroscience, IRCCS Istituto Auxologico Italiano, 20095 Cusano Milanino, MiIan Italy; 11 Dino Ferrari Center, Department of Pathophysiology and Transplantation, Center for Neurotechnology and Brain Therapeutics, Università degli Studi di Milano, Milan, Italy; 12 Department of Neurology, University of Massachusetts Medical School, Worcester, MA 02125 USA; 13 Department of Neurology, Peking University Third Hospital, 49 North Garden Road, Haidian District, Beijing, 100191 PR China; 14 Sheffield Institute for Translational Neuroscience (SITraN), University of Sheffield, Sheffield, S10 2HQ UK; 15 Rita Levi Montalcini’ Department of Neuroscience, University of Turin, Turin, Italy; 16 Neurology 1, AOU Città della Salute e della Scienza of Torino, Turin, 10124 Torino Italy; 17 Department of Neurology, Ulm University, Oberer Eselsberg 45, 89081 Ulm, Germany; 18 German Center for Neurodegenerative Diseases, DZNE, Ulm, Germany; 19 Department of Neurology, University of Ulm, Oberer Eselsberg 45, 89081 Ulm, Germany; 20 Division of Neurodegenerative Disorders, Department of Neurology, Mannheim Center for Translational Neuroscience, Medical Faculty Mannheim, Heidelberg University, Heidelberg, Germany; 21 Department Neurology, Emory University School of Medicine, Atlanta, GA 30322 USA; 22 ALS Unit, Department of Neurology, Hospital San Rafael, 28016 Madrid, Spain; 23 Neurology Department, Univeristy Hospitals Leuven, Herestraat 49, 3000 Leuven, Belgium; 24 Neuroscience Department, KU Leuven and Center for Brain & Disease Research VIB Leuven, Leuven, Belgium; 25 Complex Trait Genomics Laboratory, Smurfit Institute of Genetics, Trinity College Dublin, Dublin, D02 PN40 Ireland; 26 Academic Unit of Neurology, Trinity Biomedical Sciences Institute, Trinity College Dublin, Dublin, D02 PN40 Ireland; 27 Department of Neurology, UMC Utrecht Brain Center, University Medical Center Utrecht, Heidelberglaan 100, Utrecht, 3584 CX The Netherlands; 28 Centre de Référence pour la SLA et les Autres Maladies du Motoneurone (FILSLAN), 2 Avenue Martin Luther King, 87042 Limoges Cedex, France; 29 Centre de Compétences Neuropathies Amyloïdes Familiales et Autres Neuropathies Périphériques Rares (NNERF), Poitiers, France; 30 Neurology Clinic, Clinical Center of Serbia, School of Medicine, University of Belgrade, Studentski trg 1, Belgrade, Serbia; 31 Neuromuscular Disorders Program, Northwestern University, Feinberg School of Medicine, Chicago, IL 60208 USA; 32 Reference Center for ALS and Other Rare Motoneuron Disorders, University Hospital Gui de Chauliac, 34295 Montpellier, France; 33 Koç University, School of Medicine Translational Medicine Research Center KUTTAM-NDAL, 34450 Sarıyer, Istanbul, Turkey; 34 UK Dementia Research Institute Centre at King’s College London, School of Neuroscience, King’s College London, Strand, London, WC2R 2LS UK; 35 Centre for Brain Research, University of Auckland, 85 Park Road, Grafton, Auckland, 1023 New Zealand

**Keywords:** Medical genetics

## Abstract

*Superoxide dismutase (SOD1)* gene variants may cause amyotrophic lateral sclerosis, some of which are associated with a distinct phenotype. Most studies assess limited variants or sample sizes. In this international, retrospective observational study, we compare phenotypic and demographic characteristics between people with *SOD1*-ALS and people with ALS and no recorded *SOD1* variant. We investigate which variants are associated with age at symptom onset and time from onset to death or censoring using Cox proportional-hazards regression. The *SOD1*-ALS dataset reports age of onset for 1122 and disease duration for 883 people; the comparator population includes 10,214 and 9010 people respectively. Eight variants are associated with younger age of onset and distinct survival trajectories; a further eight associated with younger onset only and one with distinct survival only. Here we show that onset and survival are decoupled in *SOD1*-ALS. Future research should characterise rarer variants and molecular mechanisms causing the observed variability.

## Abstract (web summary)

Analysis of age of onset and disease duration in a large, international cohort of people with *SOD1*-ALS shows that there is a distinct phenotype and that onset and progression are decoupled.

## Introduction

In 1993, variants in the gene *superoxide dismutase 1 (SOD1*, [NM_000454]*)* were identified as a causal factor in people with amyotrophic lateral sclerosis (ALS), through analysis of 13 different families with 11 different *SOD1* missense mutations^1^. *SOD1* variants are reported in 15% of people with familial ALS in European populations, 30% of people with familial ALS in Asian populations, and 1–2% of people with apparently sporadic ALS in both populations^2^. Limited information is available on other populations.

*SOD1*-mediated ALS is characterised by distinct features related to the clinical and pathological phenotype. Since the discovery that variants in *SOD1* can cause ALS, over 180 variants have been identified and they are distributed throughout the gene and protein^3^. This is in contrast to other genetic determinants of ALS, for example mutations in *FUS*, *C9orf72* and *TARDBP*, where variants are concentrated in specific functional domains of the protein^4–6^. In *SOD1*-mediated ALS there is very little reported association with cognitive impairment, which, depending on cut-offs for neuropsychological deficits is estimated to occur in up to 50% of people with sporadic ALS in population-based studies^7^. People with *SOD1-*ALS are often reported to have a lower motor neuron predominant phenotype, with more frequent limb onset than is observed in typical ALS^8^. At the cellular level, TDP-43 protein aggregates, which are the pathological hallmark in >95% of ALS cases, are absent in most people with *SOD1*-mediated ALS implying that a different mechanistic pathway leads to motor neuron death^9,10^.

Within the *SOD1* ALS population, certain variants are associated with atypical disease progression compared to ALS as reported in population-based studies. For example, the p.A5V variant is associated with shorter survival and the homozygous p.D91A variant with longer survival^11,12^. Demographic factors also correlate with survival. For example, men with *SOD1-mediated* ALS have shorter survival than women^13^. Other variants, such as p.D125V and p.H44R have been associated with faster disease progression in an Australian population^14^. As gene-specific therapies for ALS are being developed it is important to understand the prognostic implications of specific variants. This was demonstrated in a trial of Tofersen, an anti-sense oligonucleotide targeting the knock down of SOD1 mRNA, where a significant impact on disease progression was noted in a subset of patients carrying the p.A5V variant, who typically have a rapid disease progression^15^.

Some variants in *SOD1* may be coincidentally found in people with ALS but not cause their disease. One way of assessing this is to compare age of symptom onset in people with *SOD1* variants and in people with sporadic ALS. In the liability threshold model of disease, a model which is consistent with ALS risk, if an individual’s liability passes a threshold, disease develops. According to the multistep model of ALS disease risk, people take on average 6 molecular steps to develop ALS, but people with *SOD1* variants need on average 2 steps—interpreted as *SOD1* variants accounting for 4 of the 6 steps^16,17^. If ALS variants increase risk of disease, we should expect them to lower the age of onset, through increasing a person’s liability to disease from birth.

To date most genotype-phenotype correlations in *SOD1*-mediated ALS are from case reports, single-centre clinic databases, and reviews. Here, we analysed the phenotypic and demographic characteristics of people with ALS with a known *SOD1* variant in a large, international dataset, to define the impact of individual variants on the age of symptom onset and survival. Understanding which variants cause the disease and their effect on the phenotype will improve genetic counselling, interpretation and application of clinical trial results and understanding of pathological mechanism.

## Results

### Case description

Once data were cleaned there were 1383 *SOD1*-ALS cases, each with a non-synonymous variant for analysis, demographic and clinical characteristics are summarised in (Table 1). Almost all records (99%), had a recorded diagnosis of ALS. The remaining 1% were recognised ALS-variants progressive muscular atrophy or primary lateral sclerosis. As the comparator dataset contained 11% of people with a diagnosis of either primary lateral sclerosis or progressive muscular atrophy, which could affect median disease duration, we ran time-to-event analyses only on those people with a recorded diagnosis of ALS according to El Escorial criteria in the comparator dataset (including all categories Definite to Suspected) and ALS without further definition in the *SOD1* dataset^26^.

**Table 1:** Demographic features of people with *SOD1* ALS

		*SOD1* dataset		Comparator dataset		*p* value (comparison)
		Total *n* = 1383	Percent	Total *n* = 12,622	Percent	
Diagnosis	ALS (incl flail limb)	1370	99	11333	89.8	<0.001 (chi squared test)
	PLS/PMA	13	1	1134	8.9	
	Not recorded	-	-	155	1.2	-
Site of onset	Spinal	1026	74.2	7957	63	<0.001 (chi squared test)
	Bulbar	108	7.8	3518	27.8	
	Mixed	8	0.58	-	-	-
	Respiratory	8	0.58	-	-	-
	Not recorded	233	16.8	1147	9.1	-
Mean age of onset years (SD)		48.9 (12.8)	NA	61.1 (12)	NA	<0.001 (t-test)
Gender	Female: Male: Not recorded	655: 726: 2	47.4: 52.5: 0.1	5137:7481:4	41:59:0.0003	<0.001 (chi squared test)
Family history	Yes: No: Not recorded	969: 185: 229	70.1: 13.4: 16.5	1853: 7016: 3753	14.3: 55.5: 28.7	<0.001 (chi squared test)
Median diagnostic delay months (IQR)		10 (19.3)	NA	12 (14)	NA	0.003 (Mann-Whitney U)
Median disease duration months (IQR)		27.7 (61.0)	NA	35.1 (35.6)	NA	<0.001 (Mann-Whitney U)
Dead	Yes: No: Unknown*	861: 284: 238	62.3: 20.5 :17.2	9108: 1677: 1837	72.2: 13.2: 14.5	<0.001 (chi squared test)

*Of the records with unknown deceased status, 48 in the SOD1 dataset and 1 in the STRENGTH dataset had disease duration data available. Two-sided tests we used to compare variables. Comparing median disease duration of people with deceased status recorded and those without this variable recorded in the SOD1 dataset showed no difference (Mann Whitney U test *p* value 0.22). These records and the comparator were excluded from the Cox proportional hazards models analysing disease duration.

*PLS* primary lateral sclerosis, *PMA* progressive muscular atrophy, *SD* standard deviation, *IQR* interquartile range.

There were 12,622 records in the comparator dataset. In both datasets, most people had limb onset ALS, but the proportions were quite different between the two, with 74% spinal onset in the *SOD1*-ALS dataset compared to 63% in the comparator dataset. Age of onset in the *SOD1*-ALS dataset was about 49 years compared to 61 years in the comparator dataset. Diagnostic delay was on average 10 months in people with *SOD1*-ALS compared 12 months for people with sporadic ALS, and survival from onset was ~28 months compared to 35 months.

**Fig. 1:**
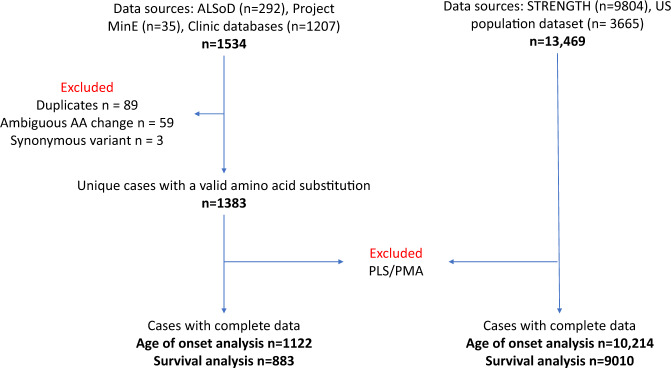
Modified CONSORT diagram of datasets included in analysis. The diagram shows the number of records identified from the following sources: ALS online Database, Project MinE, ALS Clinic databases, STRENGTH and the US population dataset. Records were excluded for missing or spurious data, or because of the diagnostic phenotype.

Of the records in the *SOD1*-ALS dataset, 1122 had complete information needed to analyse the effect of the variant on age of onset and 833 had complete data needed to analyse disease duration; the equivalent numbers in the comparator dataset were 10,214 and 9101, respectively. For more details, including which records were excluded, please see the CONSORT diagram (Fig. 1).

### Time-to-event analyses

**Fig. 2:**
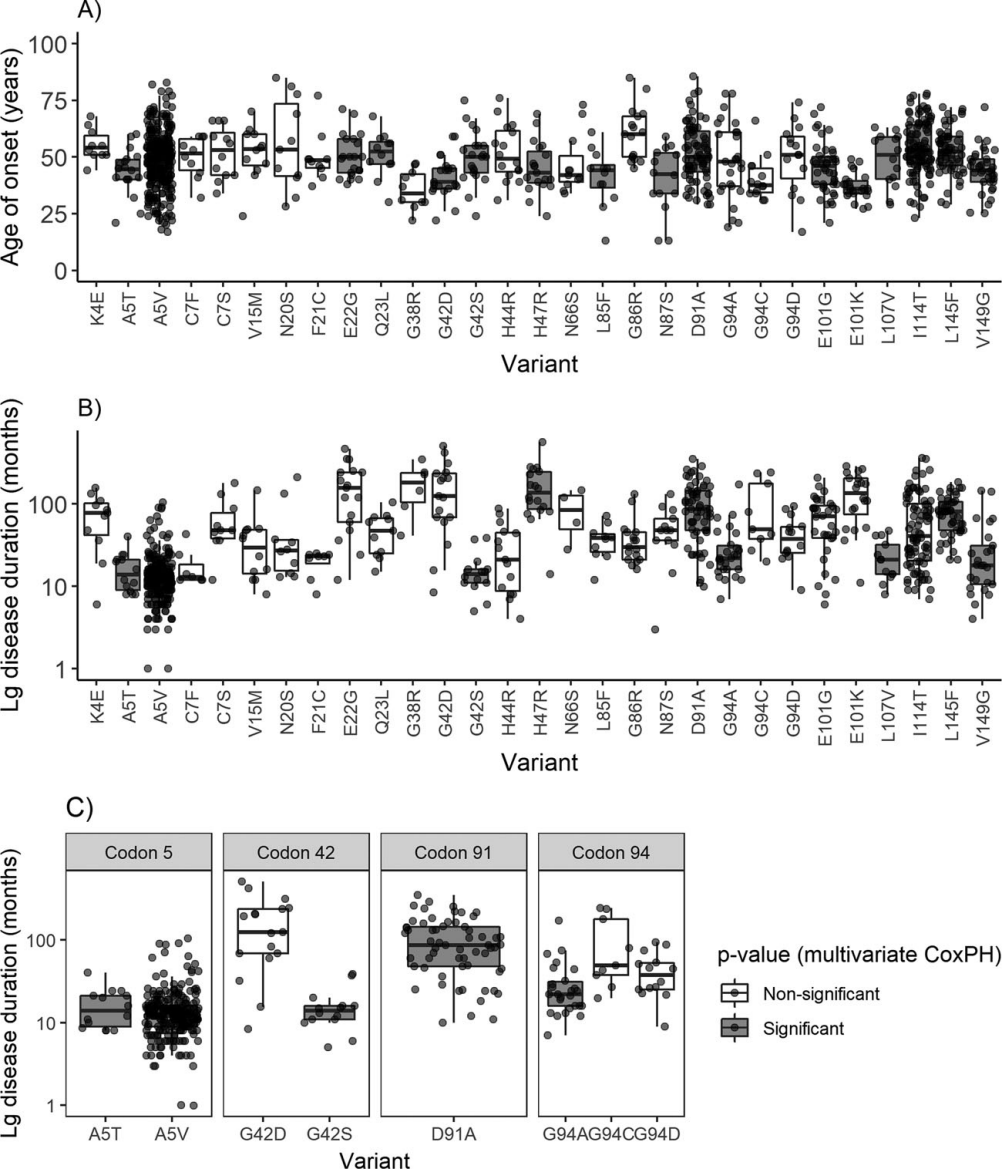
Box plots of age of onset and disease duration by variant. Information is displayed for those variants where there were >9 cases. The centre value is the median and boxes represent interquartile range with whiskers representing the minima and maxima values associated with each variant. **A** Box plot showing age of symptom onset by variant, *n* = 976. **B** Box plot of lg survival by variant, n = 809. **C** Selected box plots of log survival faceted by codon to highlight differences and similarities in survival distribution, *n* = 415. Source data are provided as a Source Data file.

**Fig. 3:**
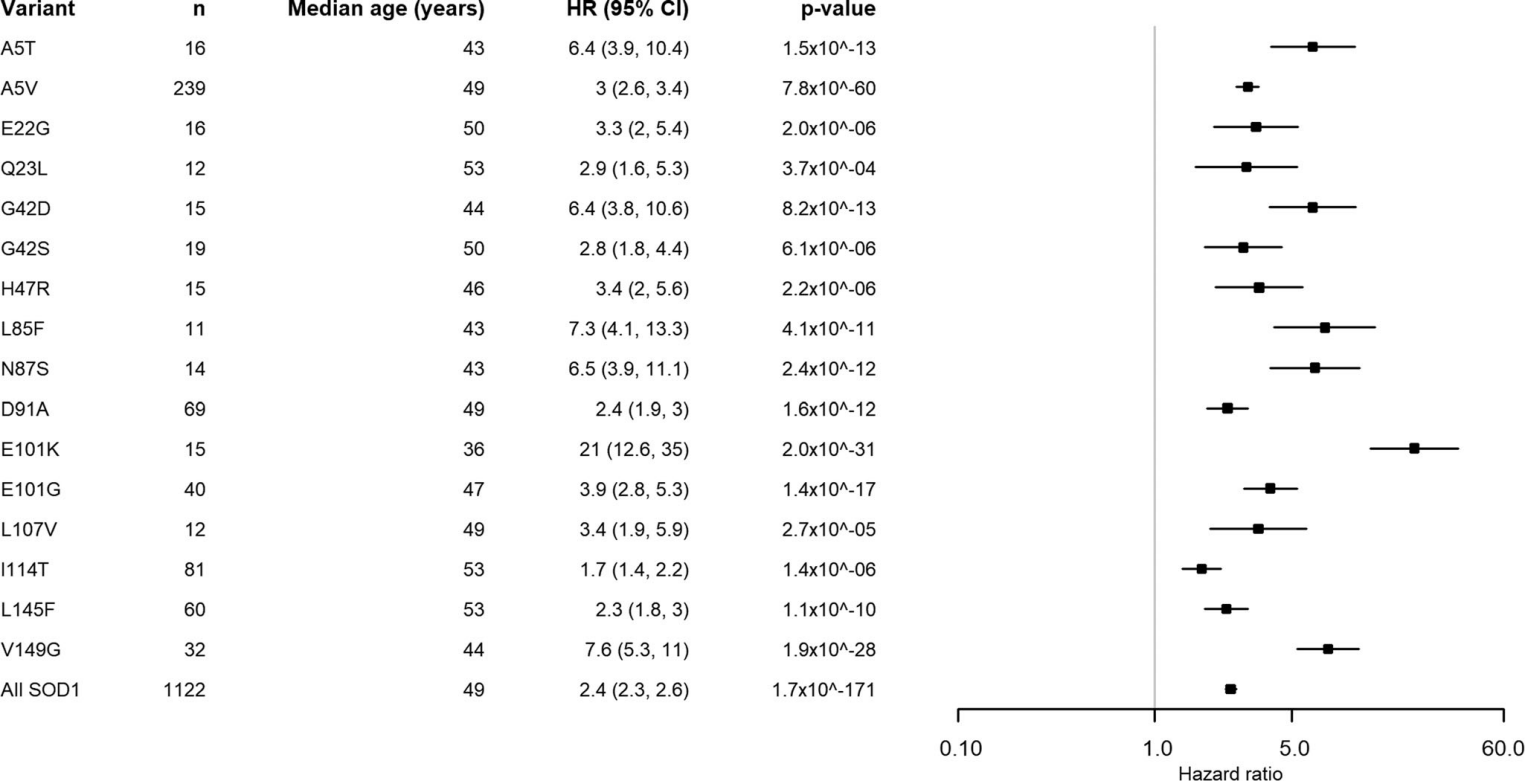
Forest plots of variants associated with age of onset. The centre of the Forest plot represents the hazard ratio of the Cox proportional hazards model, the error bars are two-sided 95% confidence intervals. All models were adjusted for site of symptom onset and gender. Source data are provided as a Source Data file.

**Fig. 4:**
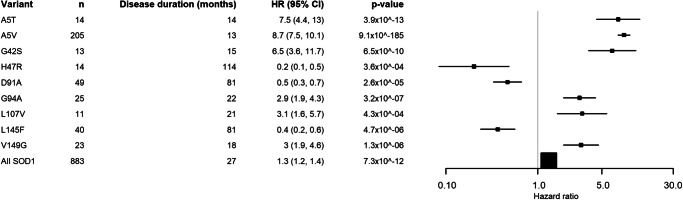
Forest plots of variants associated with disease duration. The centre of the Forest plot represents the hazard ratio of the Cox proportional hazards model, the error bars are two-sided 95% confidence intervals. All models were adjusted for site of symptoms onset, gender and age of symptom onset. Source data are provided as a Source Data file.

There was considerable variation in survival time and age of symptom onset by variant as shown in Fig. 2. The box plots include people whose survival has been right censored and are not adjusted for other factors such as site of onset. For this reason, we compared the time-to-event distributions of each individual variant where there were sufficient replicates to the population-based estimates using Cox proportional hazards regression. Variants with a *p* value smaller than the Bonferroni corrected *p* value for each Cox proportional hazards analysis and a sample size of 10 or more people per group (for each variable age of onset and disease duration) are shown in grey in Fig. 2 and are summarised in the Forest plots Figs. 3 and 4. The remainder of the results for variants with sample sizes of 9 or less are in the source data file.

Of the variants analysed with a sample size of 10 or more people, 16 variants were associated with age of onset, and 9 variants with disease duration. All strongly associated variants have a younger median age of onset than the comparator cohort. However, unlike age of onset, a third of the variants with different survival distributions had a longer survival than the typical ALS population, this is visualised in Fig. 4. Kaplan-Meier plots can be found in Supplementary Figs. S2 and S3. Of the nine variants that were associated with different survival to sporadic ALS, eight were also associated with developing the disease at a younger age. There is a lack of correlation between average age of onset of symptoms and log average disease duration as shown in Supplementary Fig. S4. All analyses of disease duration by variant included age of onset as a covariate.

**Fig. 5:**
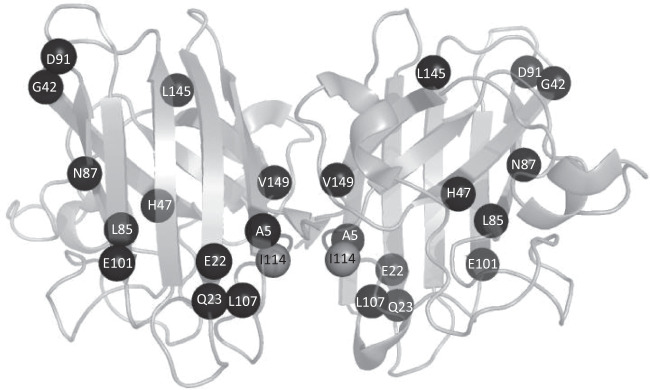
Variants associated with age of onset plotted onto a wild-type SOD1 dimer representation. Variants associated with a younger age of onset compared to the non-*SOD1* ALS population using Cox proportional hazards regression plotted onto PDB structure 2c9v. Codon numbers refer to genomic location.

**Fig. 6:**
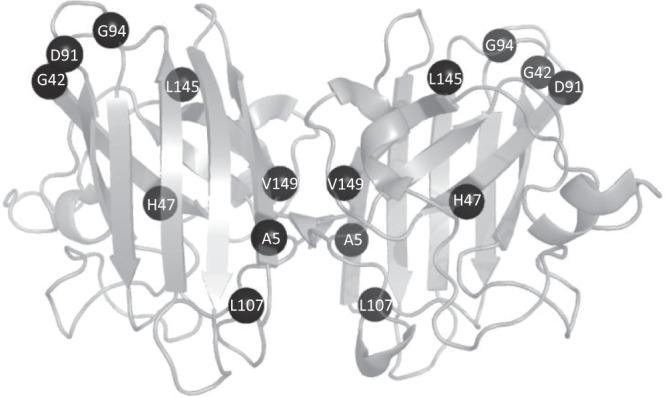
Variants associated with disease duration plotted onto a wild-type SOD1 dimer representati on. Variants associated with a distinct survival compared to the non-*SOD1* ALS population using Cox proportional hazards regression plotted onto PDB structure 2c9v. Codon numbers refer to genomic location.

Variants represented in the Forest plots are visualised on *SOD1* dimers and these are shown in Figs. 5 and 6.

Variants within the dimer interface are associated with shorter survival (HR 2.69 (95% CI 2.4, 3) *p* value = 1.28 × 10^−69 and variants in any functional domain are associated with younger age of onset when compared to the comparator dataset, which likely reflects that many SOD1 variants reduce age of onset.

## Discussion

In this study we have analysed the clinical phenotype of a large international dataset of people with ALS who have known pathogenic variants in *SOD1*. Their presentation differs from previous reports of the population-based ALS cohorts and the cohort we used as a comparator population^27^. We observed a higher percentage of limb onset ALS at 74% compared to 63% from population-based ALS cohorts and the comparator cohort. The age of symptom onset and overall survival are lower than sporadic ALS, although this is not the case when p.A5V variants are excluded from the dataset—median disease duration is longer at 45 months. There is still a slight male preponderance of 1·1 compared to 1 if all variants conformed to a mendelian autosomal dominant pattern of inheritance. Given that *SOD1* variants account for four of the six presumed steps taken to develop ALS (according to the multistep model of ALS in population-based cohorts and subsequent recalculations in genetic subtypes) the other steps may be related to risks that men are exposed to more than women^17^. Most people in the dataset have a family history of ALS but about 10% are listed as being apparently sporadic. De novo mutations in *SOD1* are rare and the absence of a family history is more likely to be due to incomplete penetrance, inadequate record keeping or small family size, which may mask familiality, supported by identity by descent analysis that has identified familial links in apparently sporadic *SOD1* ALS^28–30^.

We have compared the age of symptom onset and survival in *SOD1*-ALS to a population-based comparator cohort to identify variants that statistically significantly affected the clinical phenotype (i.e. had *p* values lower than the respective multiple-testing thresholds of 0.0007 and 0.00082). Of the 16 variants (with group size >9) associated with age of onset, 8 were also associated with survival, some of which had a longer median survival. This apparent uncoupling of age of symptom onset and survival after symptom onset suggests that different mechanisms are involved in the initiation of neurodegeneration and its progression for the majority of variants, a finding supported by a recent genome-wide association analysis of ALS^31^. A similar uncoupling between age of symptom onset and progression is seen in Huntington’s disease. Increased CAG repeat length and polyglutamine tract of the Htt protein is strongly correlated with an earlier age of onset of motor symptoms but not the rate of disease progression and survival^32^. It has been postulated that this is either because onset and death are due to damage to different cell types which the variant affects differently or, that the variant damages cells, causing disease onset followed by a variant-independent process that then leads to cell death. Either of these scenarios could be relevant to ALS as the toxicity of mutant SOD1 and Htt are due to protein misfolding and may be true for other genetic neurodegenerative disorders.

Sixty-six of the 70 variants analysed for differences in age of onset had a younger median age of onset than people with ALS in the comparator dataset, and none of the four with an older median age of onset passed the multiple testing threshold p-value, although the sample sizes in these groups were five people or fewer. Interpreted in the context of the multistep and liability threshold models of disease, both of which are consistent with ALS risk, this trend, along with the high number of variants passing the multiple-testing threshold implies that the variants are likely to be risk variants rather than being randomly found in people with ALS, although this study does not replace other epidemiological methods. 70% of people in the *SOD1*-ALS dataset had a family history of ALS, however earlier recorded age of onset is not likely to be due to ascertainment bias as people with sporadic ALS with a high genetic liability have younger age of onset^33^. In the *SOD1* dataset there is no difference in age of onset between people a positive family history and those with a negative family history HR 1.01 (95% CI 0.83, 1.24), *p* value = 0.9. As people with a *SOD1* variant but with a negative family history can be considered people with a high genetic liability, this supports those findings. Inheritance patterns in ALS have been reported as Mendelian, polygenic and oligogenic and it is possible that variants in different parts of the *SOD1* gene cause risk through each of these various patterns^34^. Variants in *SOD1* could contribute to polygenic or oligogenic risk and this may be related to differing TDP-43 pathology in some cases of *SOD1-*ALS.

In contrast to the frequent observation of a relationship between *SOD1* mutation and reduced age of onset, only a few variants differentially influenced survival, and a third of those appeared to be protective, lengthening rather than shortening survival. The variants most strongly associated with shorter disease duration tended to be closer to the N-terminus of the protein (at positions 5, 7, 21 and 42), which may relate to the amyloid core of both wild-type and mutant SOD1 fibrils being located towards the N-terminus of the protein but there were exceptions^35^. A change from glycine to serine at codon 42 was associated with longer disease duration but a change from glycine to aspartic acid was not, this is in stark contrast with variants at codon 5, which were both associated with shorter survival. At codon 7, only C7G was associated with shorter disease duration, despite all three variants at codon 7 being associated with younger disease onset, and similarly at codon 94 only G94A was associated with shorter disease duration and not younger age of onset whereas G94C and G94D were associated with younger age of onset and not disease duration. There is likely an interaction between location, the nature of the amino acid substitution and the location in the protein structure, and subsequent effect on the thermodynamic stability of the SOD1 dimer, the creation of additional fibril-forming seed regions or alteration of packing around the existing fibril cores, and other factors as yet undetermined. Solving this problem was beyond the scope of this study, but we hope that the data presented here will aid in the design of further experimental and in silico studies to identify such complex correlations.

Understanding which factors cause disease and which affect clinical progression will improve genetic counselling and development of therapies. There are currently clinical trials using *SOD1* antisense oligonucleotides and the largest effects may be observed in the variants with the shortest survival time.^15^ It is not clear what impact therapy will have on people carrying variants associated with a longer disease duration, although if effective, the therapy should halt progression altogether. However, for people with slow-progression variants, the effect of gene therapy may be more difficult to prove without lengthy observation. In trials of lithium in ALS, there was a survival benefit in people with variants in the *UNC13A* gene, and this brought them in line with ALS survival in the control group without *UNC13A* variants^36^. Analysing subgroups based on faster progressing variants may be appropriate, and useful for people before they receive therapy to understand better their survival benefit.

The limitations of this study are that it is mainly based on clinic populations or single case studies and a large proportion of the dataset is made up of people from the US with p.A5V variants which may not be generalisable to the global *SOD1*-ALS population. This underscores the need to characterise phenotype by variant in analyses. The clinical data for people with *SOD1-*ALS are limited in scope and there is missing data. In our survival modelling, the comparator population is European-derived and does not represent a comparator dataset for all of the countries represented in the *SOD1* dataset. As a sensitivity analysis we have run our main analysis, restricting the *SOD1* dataset to only those countries represented in the comparator dataset. Although this reduces the number of variants available for analysis our conclusions remain the same in that there are more variants associated with younger age of onset than shorter survival and many of the same variants have strong associations with both outcome measures. There may be people represented in both datasets, although this is more likely for UK and Italian people as there were not many people with *SOD1-*ALS in Irish, Dutch and Belgian populations, and it is at most a very small proportion of the comparator dataset. We plan to develop a web-tool using the dataset from this study so people can use comparator populations they feel are appropriate when analysing their own data.

We have characterised the effect of a number of *SOD1* variants on the ALS phenotype but, some *SOD1* variants are very rare and a larger number of ALS patients harbouring those variants is needed to study them. Additional work is needed to characterise the molecular mechanism behind this variability of effect on the clinical phenotype.

## Methods

### Data sources

The data analysed in this project were either in the public domain (phenotype information sources from scientific publications) or were fully anonymised at source and therefore completely anonymous at the point of access. No new data were collected for this study. Following King’s College London Research Governance protocols ethical clearance was not required for this study.

#### SOD1 cohort

We primarily accessed the ALS Online Database, a manually curated collection of published evidence about genes and genetic variants associated with ALS (https://alsod.ac.uk)^18^. The database includes clinical data collected from individual or family case reports of 150 genes including *SOD1*, with data available at variant level. In the instance of missing data, corresponding authors were contacted to ask for further information. We also contacted clinicians working in specialist centres that performed genetic testing and requested they provide anonymised records of people with *SOD1*-ALS. Each data source and their local ethical approval are detailed below:

Macquarie University: participants recruited under informed written consent as approved by the Human Research Ethics Committee of Macquarie University.

ANZAC Research Institute: participants recruited under informed written consent as approved by the institutional review board of the ANZAC Research Institute (Sydney South West Area Health Service).

University of Massachusetts: data were acquired with formal patient consent according to protocols reviewed and approved by the Institutional Review Boards of first the Massachusetts General Hospital and then the University of Massachusetts Medical School.

University Hospitals of Montpellier: all participants consented for storage of their data and its use in research, the study was approved by the Ethics committee (CCPPRB) of Pitié Salpêtrière Hospital n°131/92.

King’s College London: participants provided consent for storage of their genetic and clinical data and its use for research in protocols approved by Local Research Ethics Committee approval number 222/02.

Washington University School of Medicine in St Louis: the data was collected under a waiver of consent since the participants were all deceased.

Peking University Third Hospital: all patients included provided written informed consent to participate in the clinical and genetic studies, which were approved by the institutional ethics committee of Peking University Third Hospital (PUTH).

Northwestern Medicine – Feinberg School of Medicine: Northwestern’s Institutional Review Board has reviewed and approved our Neurological Diseases Registry annually since 1991. Consents include the statement that data obtained from studying the subject’s contributions may be shared with other researchers as long as the data is deidentified.

Istituto Auxologico Italiano IRCCS-University of Milan: data were collected in the project SOD1-ITALS approved by Ethical Committee of the IRCCS Istituto Auxologico Italiano.

University of Belgrade: All individuals gave written informed consent for the storage of their data and its use in research and the Ethics Committee of the School of Medicine at the University of Belgrade approved this protocol.

Koç University: data and sample collection was approved by Boğaziçi University Ethics Committee. Signed informed consent was obtained from all subjects. The storage of the data and its use for research was approved by the patients.

Project MinE: the Project MinE database was searched for people with ALS in whom *SOD1* variants had been identified by whole genome sequencing the ethical approval for the project MinE dataset is described in detail elsewhere^19^.

#### Comparator cohort

To compare age of onset and survival in people from the general ALS population and *SOD1*-mediated ALS we used a comparator population of people from population-based datasets of ALS in five European populations (UK, Netherlands, Italy, Ireland and Belgium) and the United States. The data from European countries consisted of clinical variables only that were originally collected and analysed as part of the Survival, Trigger and Risk, Epigenetic, eNvironmental and Genetic Targets for motor neuron Health (STRENGTH) project. The ethical approval for the European and US datasets are described in detail elsewhere^19,20^.

Additionally, countries included in the study are visualised in Supplementary Fig. S1.

### Clinical and demographic variables—SOD1 dataset

People were eligible if they had a recorded diagnosis of ALS made by a Consultant Neurologist, or their diagnosis was published as ALS in the literature. Two people were described as having ALS-flail limb, and these were coded as ALS. We collected sex at birth and age of onset (in years) of first motor symptoms of ALS, defined as first weakness or speaking or swallowing difficulty. Site of onset was coded as bulbar, spinal, respiratory or mixed. We asked whether people had a family history of ALS as reported by their clinician with no specific definition. To record disease progression, we collected or requested the time in months from onset of motor symptoms to diagnosis as well as the months onset to death, or their most recent appointment date. Additionally, we asked whether the person was deceased or not as a binary variable, and where this was missing, we coded it as the person not being deceased. Finally, we asked whether the person had been diagnosed with dementia; this was not specified as being a formal diagnosis of frontotemporal dementia. As data were fully anonymised, we were not able to use personal identifiers to find duplicate records. Records from different sources but with the same variant, country of origin, gender, age of onset and site of onset were assumed to be duplicates.

### Genetic variants

Amino acid change was denoted by genomic location (rather than the historic notation that not including the initial methionine, for example we used p.A5V/p.D91A rather than A4V/D90A). In some cases, this can lead to ambiguity. For example, position 113 and 114 in this format are both isoleucine, so that I114 could refer to I113 or I114 if the format is not specified. In such situations, the original source was checked for how it referred to other non-ambiguous variants, so for example if a case study source referred to someone as having an A4V variant and someone else as an I113T we assumed they were using the format not including the initial methionine recoded the ambiguous variant as p.I114T. Where it was not possible to determine which format the variant referred to, if the variant was impossible given the DNA sequence at that codon and it could not be clarified, or if the source referred to a general location, for example just recording an insertion into an exon with no further details, these were classed as ‘ambiguous’ and excluded. Additionally, we did not analyse data on synonymous variants. For readability ‘p.’ has been left off the graphs when referencing a variant.

### Functional location of genetic variants

Amino acids that are within 6 Å of the dimer interface were classed as being within the dimer interface. The codons making up the electrostatic loop and dimer interface were defined according to those amino acids identified as being in those areas according to the literature^21^. If the codon was in the dimer interface and the electrostatic loop or the zinc loop, they were classified in those locations rather than in the dimer interface.

The codon numbers and their corresponding location are:

Dimer interface: 4–10, 18–20, 50–55, 60–62, 112–116, 148–154

Electrostatic loop: 122–134

Zinc loop: 51–84

Pymol version 1.7.1.1 was used to plot variants associated with age of onset and disease duration onto PDB structure 9c2v.

### Statistical analysis

Frequencies of phenotype groups in the *SOD1* dataset were compared with those in the population-based ALS comparator dataset using descriptive statistics.

Time-to-event analysis to assess which variants are strongly associated with age of onset and disease duration from onset was performed using Cox proportional hazards regression for variants found in three or more people. Due to the unreliability of Cox proportional hazards regression at smaller sample sizes only those with 10 or more cases are displayed in the main manuscript, the remainder can be found in the source data file. Models were adjusted for site of onset of symptoms and gender the coxph() function was utilised with tie resolution at the default setting. In addition we calculated Cohen’s *D* for goodness of fit using the royston() function with default settings. When modelling disease duration from onset, age of onset was also included as a covariate. Modelling by functional location of variants was performed separately but the covariates were the same for each outcome.

There were 70 variants eligible for time-to-event analysis modelling for age of onset and 61 available for time-to-event analysis of survival; the Bonferroni corrected p-value thresholds for these analyses were 0.0007 and 0.00086, respectively. Data were analysed and visualised in R version 4.0.2 using the packages ‘ggplot2’ (version 3.3.5) ‘rworldmap’ (version 1.3-6) and ‘survival’ (version 3.2-7)^22–25^.

### Role of the funding source

The study sponsors were not involved in the study design, collection, analysis, interpretation of data or in the writing of the report.

### Reporting summary

Further information on research design is available in the Nature Portfolio Reporting Summary linked to this article.

### Supplementary information

Supplementary Information

Reporting Summary

### Source data

Source data

## Data Availability

The raw data used for this study are available under restricted access as per agreement with data contributors and are available on request by contacting alsod@kcl.ac.uk. De-identified data will be made available within 6 weeks of request. Summary level data generated by Cox proportional hazards modelling are available in source data file and at sod1-alsphen.rosalind.kcl.ac.uk. Source data used to generate Figs. 2–4 are provided in the Source data file. Source data are provided with this paper.

## Code availability

The code used for the Cox proportional hazards regression and Figs. 2–4 is available at https://github.com/opiemartin/SOD1-survival.

## References

[1] Rosen DR (1993). Mutations in Cu/Zn superoxide dismutase gene are associated with familial amyotrophic lateral sclerosis. Nature.

[2] Zou Z-Y (2017). Genetic epidemiology of amyotrophic lateral sclerosis: a systematic review and meta-analysis. J. Neurol., Neurosurg. Psychiatry.

[3] Shaw CE (1998). Mutations in all five exons of SOD-1 may cause ALS. Ann. Neurol..

[4] Daoud H (2012). C9orf72 hexanucleotide repeat expansions as the causative mutation for chromosome 9p21–linked amyotrophic lateral sclerosis and frontotemporal dementia. Arch. Neurol..

[5] Deng H, Gao K, Jankovic J (2014). The role of FUS gene variants in neurodegenerative diseases. Nat. Rev. Neurol..

[6] Mackenzie IRA, Rademakers R (2008). The role of transactive response DNA-binding protein-43 in amyotrophic lateral sclerosis and frontotemporal dementia. Curr. Opin. Neurol..

[7] Wicks P (2009). SOD1 and cognitive dysfunction in familial amyotrophic lateral sclerosis. J. Neurol..

[8] Li H-F, Wu Z-Y (2016). Genotype-phenotype correlations of amyotrophic lateral sclerosis. Transl. Neurodegener..

[9] Lanznaster D, Hergesheimer R, Vourc’h P, Corcia P, Blasco H (2021). TDP43 aggregates: the ‘Schrödinger’s cat’ in amyotrophic lateral sclerosis. Nat. Rev. Neurosci..

[10] Tziortzouda P, Van Den Bosch L, Hirth F (2021). Reply to ‘TDP43 aggregates: the ‘Schrödinger’s cat’ in amyotrophic lateral sclerosis’. Nat. Rev. Neurosci..

[11] Bali T (2017). Defining SOD1 ALS natural history to guide therapeutic clinical trial design. J. Neurol. Neurosurg. Psychiatry.

[12] Parton MJ (2002). D90A-SOD1 mediated amyotrophic lateral sclerosis: a single founder for all cases with evidence for a Cis-acting disease modifier in the recessive haplotype. Hum. Mutat..

[13] Tang L., Ma Y., Liu X.-L., Chen L. & Fan D.-S. Better survival in female SOD1-mutant patients with ALS: a study of SOD1-related natural history. *Transl. Neurodegener.*;**8**:2 (2019). 10.1186/s40035-018-0142-8 PMC6325854 30637102

[14] McCann EP (2017). The genotype–phenotype landscape of familial amyotrophic lateral sclerosis in Australia. Clin. Genet..

[15] Miller T., et al. Phase 1–2 trial of antisense oligonucleotide tofersen for SOD1 ALS. N. Engl. J. Med. **383**: 109–119 (2020). 10.1056/NEJMoa2003715 32640130

[16] Al-Chalabi A (2014). Analysis of amyotrophic lateral sclerosis as a multistep process: a population-based modelling study. Lancet Neurol..

[17] Chiò A (2018). The multistep hypothesis of ALS revisited: the role of genetic mutations. Neurology.

[18] Abel O, Powell JF, Andersen PM, Al-Chalabi A (2012). ALSoD: a user-friendly online bioinformatics tool for amyotrophic lateral sclerosis genetics. Hum. Mutat..

[19] Van Rheenen W (2018). Project MinE: study design and pilot analyses of a large-scale whole-genome sequencing study in amyotrophic lateral sclerosis. Eur. J. Hum. Genet..

[20] Shatunov A (2010). Chromosome 9p21 in sporadic amyotrophic lateral sclerosis in the UK and seven other countries: a genome-wide association study. Lancet Neurol..

[21] Galaleldeen A (2009). Structural and biophysical properties of metal-free pathogenic SOD1 mutants A4V and G93A. Arch. Biochem. Biophys..

[22] South A (2011). rworldmap: a new R package for mapping global. Data. R. J..

[23] Team R. C. R.: A Language and Environment for Statistical Computing. *R Foundation for Statistical Computing* 2020.

[24] Therneau T. M. A Package for Survival Analysis in R. 2021.

[25] Wickham H. ggplot2: Elegant Graphics for Data Analysis: Springer-Verlag New York; 2016.

[26] Brooks BR, Miller RG, Swash M, Munsat TL (2000). El Escorial revisited: revised criteria for the diagnosis of amyotrophic lateral sclerosis. Amyotroph. Lateral Scler. Other Motor. Neuron. Disord..

[27] Marin B (2016). Clinical and demographic factors and outcome of amyotrophic lateral sclerosis in relation to population ancestral origin. Eur. J. Epidemiol..

[28] Al-Chalabi A, Lewis CM (2011). Modelling the effects of penetrance and family size on rates of sporadic and familial disease. Hum. Hered..

[29] Henden L (2020). Identity by descent analysis identifies founder events and links SOD1 familial and sporadic ALS cases. npj Genom. Med..

[30] Ryan M., et al. Determining the incidence of familiality in ALS: a study of temporal trends in Ireland from 1994 to 2016. *Neurol. Genet.* **4**: e239-e (2018). 10.1212/NXG.0000000000000239 PMC5961194 29845113

[31] van Rheenen W (2016). Genome-wide association analyses identify new risk variants and the genetic architecture of amyotrophic lateral sclerosis. Nat. Genet..

[32] Keum JW (2016). The HTT CAG-expansion mutation determines age at death but not disease duration in huntington disease. Am. J. Hum. Genet.

[33] Mehta PR (2019). Younger age of onset in familial amyotrophic lateral sclerosis is a result of pathogenic gene variants, rather than ascertainment bias. J. Neurol. Neurosurg. Psychiatry.

[34] Veldink JH (2017). ALS genetic epidemiology ‘How simplex is the genetic epidemiology of ALS?’. J. Neurol. Neurosurg. Psychiatry.

[35] Chan PK (2013). Structural similarity of wild-type and ALS-mutant superoxide dismutase-1 fibrils using limited proteolysis and atomic force microscopy. Proc. Natl Acad. Sci..

[36] van Eijk RPA (2017). Meta-analysis of pharmacogenetic interactions in amyotrophic lateral sclerosis clinical trials. Neurology.

