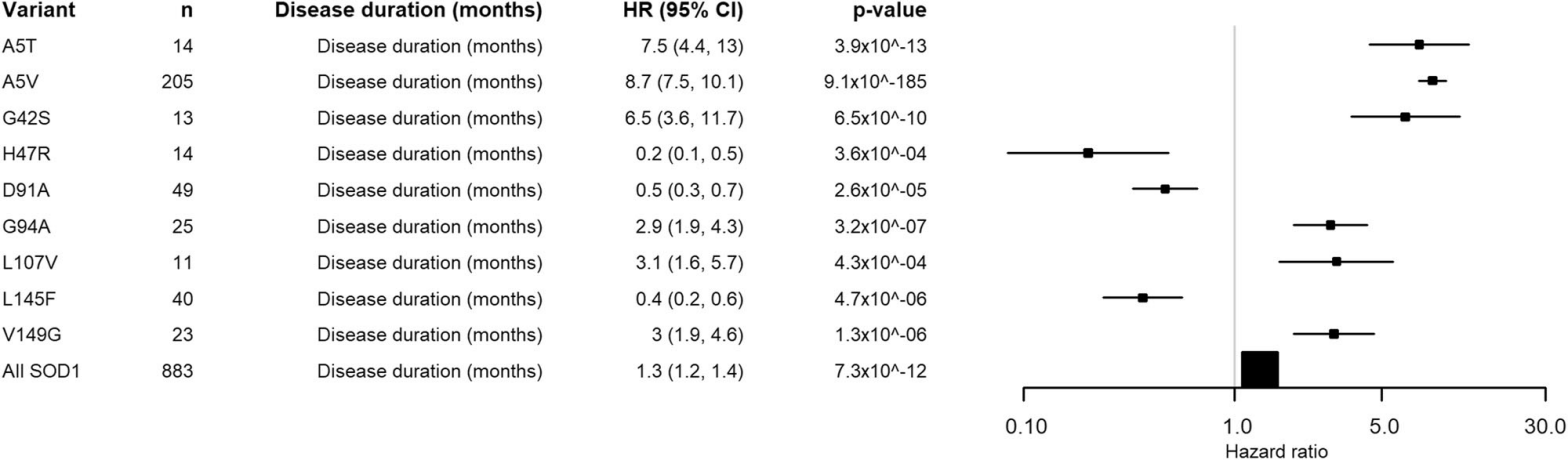# Author Correction: The *SOD1*-mediated ALS phenotype shows a decoupling between age of symptom onset and disease duration

**DOI:** 10.1038/s41467-024-49938-y

**Published:** 2024-07-02

**Authors:** Sarah Opie-Martin, Alfredo Iacoangeli, Simon D. Topp, Olubunmi Abel, Keith Mayl, Puja R. Mehta, Aleksey Shatunov, Isabella Fogh, Harry Bowles, Naomi Limbachiya, Thomas P. Spargo, Ahmad Al-Khleifat, Kelly L. Williams, Jennifer Jockel-Balsarotti, Taha Bali, Wade Self, Lyndal Henden, Garth A. Nicholson, Nicola Ticozzi, Diane McKenna-Yasek, Lu Tang, Pamela J. Shaw, Adriano Chio, Albert Ludolph, Jochen H. Weishaupt, John E. Landers, Jonathan D. Glass, Jesus S. Mora, Wim Robberecht, Philip Van Damme, Russell McLaughlin, Orla Hardiman, Leonard van den Berg, Jan H. Veldink, Phillippe Corcia, Zorica Stevic, Nailah Siddique, Vincenzo Silani, Ian P. Blair, Dong-sheng Fan, Florence Esselin, Elisa de la Cruz, William Camu, Nazli A. Basak, Teepu Siddique, Timothy Miller, Robert H. Brown, Ammar Al-Chalabi, Christopher E. Shaw

**Affiliations:** 1 Department of Basic and Clinical Neuroscience, Maurice Wohl Clinical Neuroscience Institute, Institute of Psychiatry, Psychology and Neuroscience, King’s College London, London, SE5 9NU UK; 2 Department of Biostatistics and Health Informatics, Institute of Psychiatry Psychology & Neuroscience, King’s College London, SE5 8AF London, UK; 3 NIHR Biomedical Research Centre at South London and Maudsley NHS Foundation Trust and King’s College London, London, UK; 4 Homerton University Hospital, Homerton Row, London, E9 6SR UK; 5 Department of Molecular and Clinical Pharmacology, University of Liverpool, Blue Block 1.09, Sherrington Building, Crown St, Liverpool, L693BX UK; 6 Institute of Medicine, North-Eastern Federal University, 58 Belinsky str, Yakutsk, 677000 Russia; 7 Macquarie University Centre for MND Research, Macquarie Medical School, Faculty of Medicine, Health and Human Sciences, Macquarie University, Sydney, NSW Australia; 8 Department of Neurology, Washington University School of Medicine, St Louis, MO 63110 USA; 9 Concord Clinical School, ANZAC Research Institute, Concord Repatriation Hospital, Sydney, NSW 2139 Australia; 10 Department of Neurology and Laboratory of Neuroscience, IRCCS Istituto Auxologico Italiano, 20095 Cusano Milanino, MiIan Italy; 11 Dino Ferrari Center, Department of Pathophysiology and Transplantation, Center for Neurotechnology and Brain Therapeutics, Università degli Studi di Milano, Milan, Italy; 12 Department of Neurology, University of Massachusetts Medical School, Worcester, MA 02125 USA; 13 Department of Neurology, Peking University Third Hospital, 49 North Garden Road, Haidian District, Beijing, 100191 PR China; 14 Sheffield Institute for Translational Neuroscience (SITraN), University of Sheffield, Sheffield, S10 2HQ UK; 15 Rita Levi Montalcini’ Department of Neuroscience, University of Turin, Turin, Italy; 16 Neurology 1, AOU Città della Salute e della Scienza of Torino, Turin, 10124 Torino Italy; 17 Department of Neurology, Ulm University, Oberer Eselsberg 45, 89081 Ulm, Germany; 18 German Center for Neurodegenerative Diseases, DZNE, Ulm, Germany; 19 Department of Neurology, University of Ulm, Oberer Eselsberg 45, 89081 Ulm, Germany; 20 Division of Neurodegenerative Disorders, Department of Neurology, Mannheim Center for Translational Neuroscience, Medical Faculty Mannheim, Heidelberg University, Heidelberg, Germany; 21 Department Neurology, Emory University School of Medicine, Atlanta, GA 30322 USA; 22 ALS Unit, Department of Neurology, Hospital San Rafael, 28016 Madrid, Spain; 23 Neurology Department, Univeristy Hospitals Leuven, Herestraat 49, 3000 Leuven, Belgium; 24 Neuroscience Department, KU Leuven and Center for Brain & Disease Research VIB Leuven, Leuven, Belgium; 25 Complex Trait Genomics Laboratory, Smurfit Institute of Genetics, Trinity College Dublin, Dublin, D02 PN40 Ireland; 26 Academic Unit of Neurology, Trinity Biomedical Sciences Institute, Trinity College Dublin, Dublin, D02 PN40 Ireland; 27 Department of Neurology, UMC Utrecht Brain Center, University Medical Center Utrecht, Heidelberglaan 100, Utrecht, 3584 CX The Netherlands; 28 Centre de Référence pour la SLA et les Autres Maladies du Motoneurone (FILSLAN), 2 Avenue Martin Luther King, 87042 Limoges Cedex, France; 29 Centre de Compétences Neuropathies Amyloïdes Familiales et Autres Neuropathies Périphériques Rares (NNERF), Poitiers, France; 30 Neurology Clinic, Clinical Center of Serbia, School of Medicine, University of Belgrade, Studentski trg 1, Belgrade, Serbia; 31 Neuromuscular Disorders Program, Northwestern University, Feinberg School of Medicine, Chicago, IL 60208 USA; 32 Reference Center for ALS and Other Rare Motoneuron Disorders, University Hospital Gui de Chauliac, 34295 Montpellier, France; 33 Koç University, School of Medicine Translational Medicine Research Center KUTTAM-NDAL, 34450 Sarıyer, Istanbul, Turkey; 34 UK Dementia Research Institute Centre at King’s College London, School of Neuroscience, King’s College London, Strand, London, WC2R 2LS UK; 35 Centre for Brain Research, University of Auckland, 85 Park Road, Grafton, Auckland, 1023 New Zealand

**Keywords:** Medical genetics

Correction to: *Nature Communications* 10.1038/s41467-022-34620-y, published online 12 November 2022

The original version of this Article contained an error in Figure 4. Under the heading ‘Disease duration (months)’, all disease durations were reported as ‘disease duration’ rather than reporting the numerical value. The correct version now reports the disease duration in months instead of the original, incorrect ‘disease duration’. This has been corrected in both the PDF and HTML versions of the Article.

The correct version of Figure 4 is:
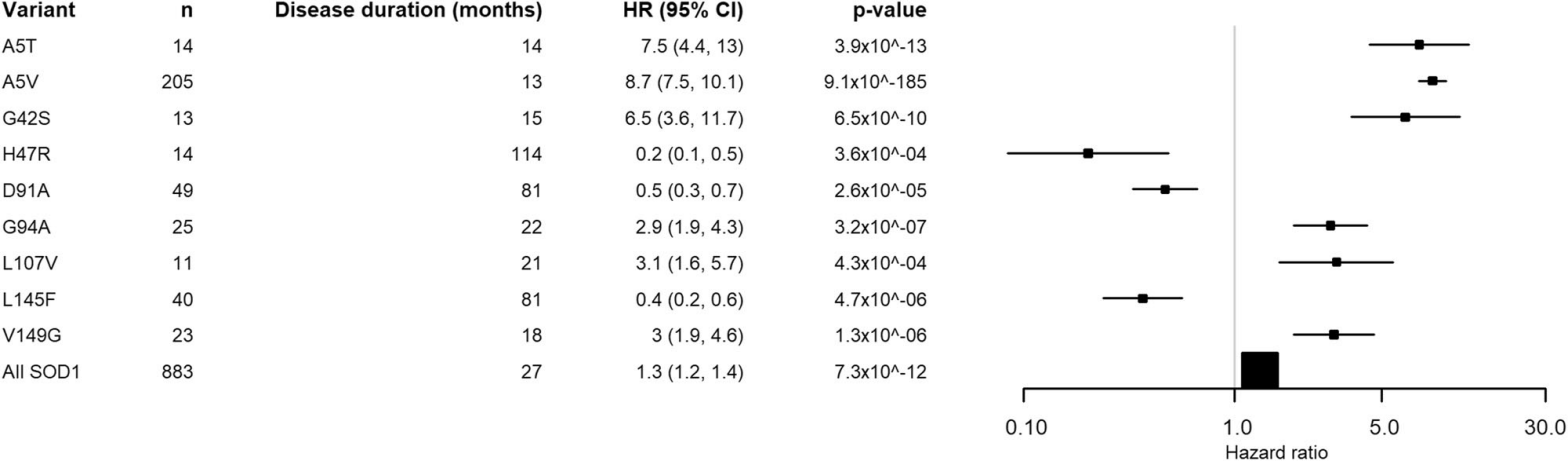


which replaces the previous incorrect version: