# Supplementary material for: The SOD1-mediated ALS phenotype shows a decoupling between age of symptom onset and disease duration
Source: Nat Commun. 2022 Nov 12;13:6901. doi: 10.1038/s41467-022-34620-y (PMC9653399; doi:10.1038/s41467-022-34620-y)
Supplement: Supplementary file 1 — Supplementary Information [file 41467_2022_34620_MOESM1_ESM.pdf]

The *SOD1*-mediated ALS phenotype shows a decoupling between age of symptom onset and disease duration

Supplementary information

Table of Contents

Supplementary figures 2

Supplementary figures

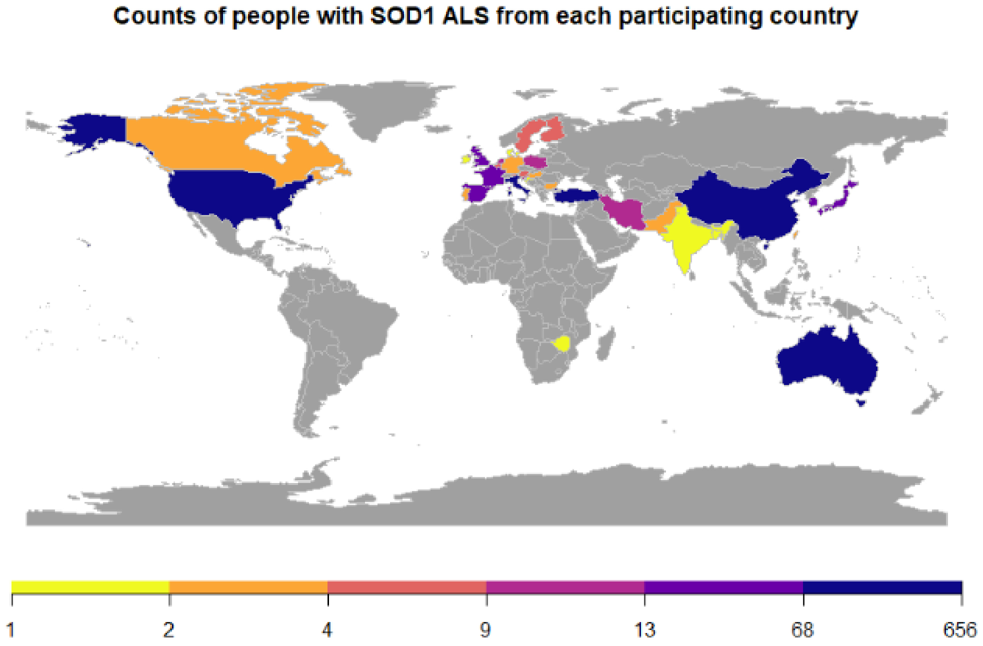

Figure S1 World map showing the 31 countries in the cleaned dataset (n=1534). The numbers of people per country in the dataset are represented by sextiles.

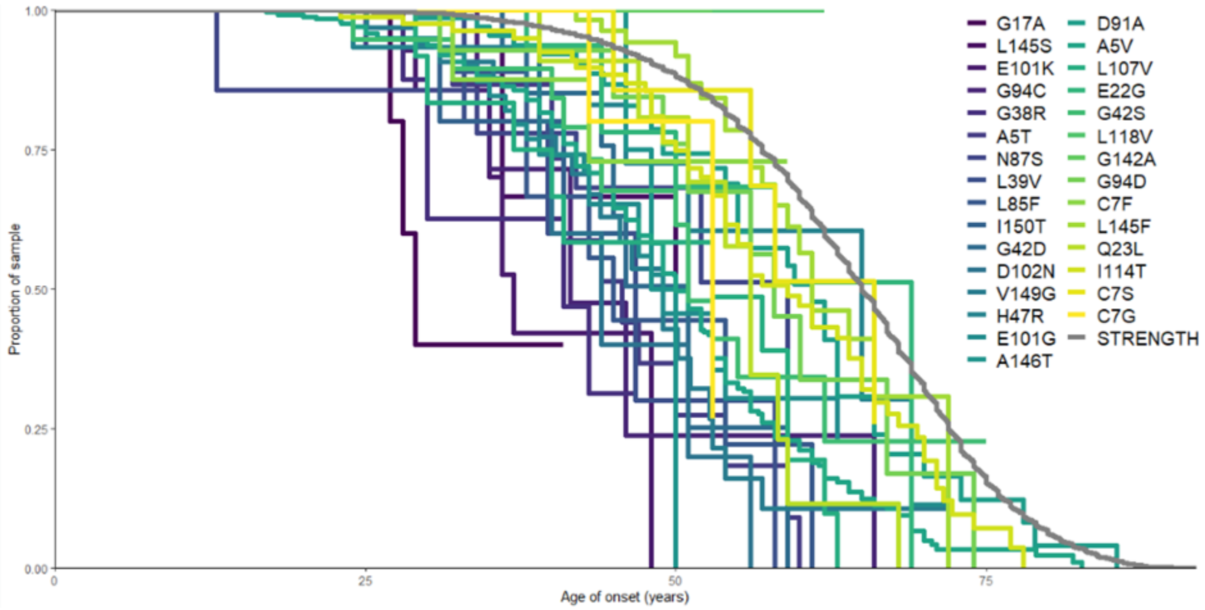

Figure S2 Kaplan-Meier plot showing time to onset of disease in people with different SOD1 variants compared to the STRENGTH cohort.

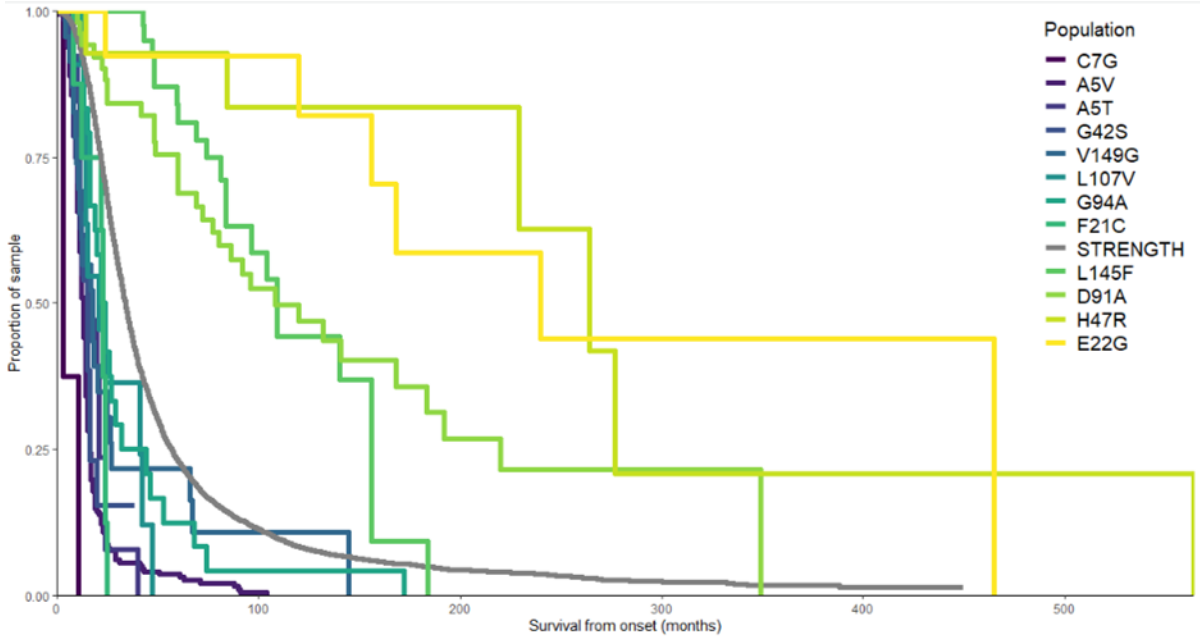

Figure S3 Kaplan-Meier plot showing disease duration in people with different SOD1 variants compared to the STRENGTH cohort.

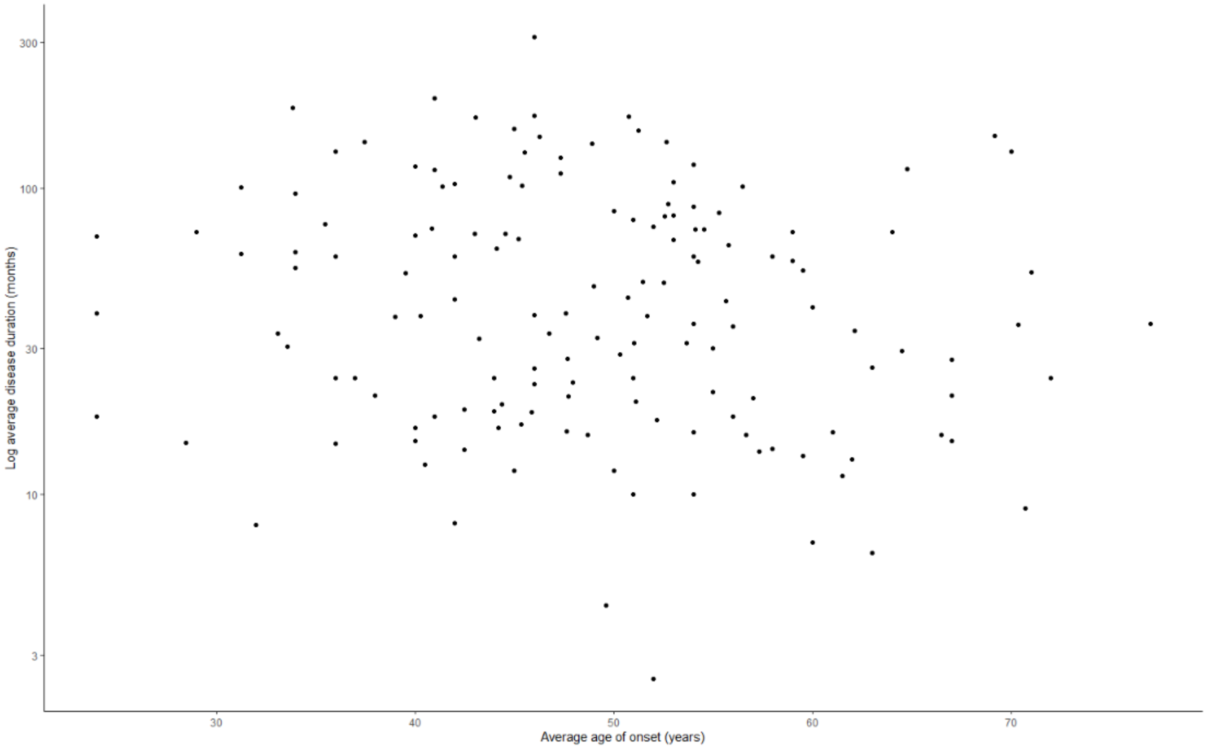

Figure S4 Scatter plot of average age of onset and log average disease duration. Each dot represents a SOD1 variant.
