# Supplementary material for: The SOD1-mediated ALS phenotype shows a decoupling between age of symptom onset and disease duration
Source: Nat Commun. 2022 Nov 12;13:6901. doi: 10.1038/s41467-022-34620-y (PMC9653399; doi:10.1038/s41467-022-34620-y)
Supplement: Supplementary file 2 — Reporting Summary [file 41467_2022_34620_MOESM2_ESM.pdf]

nature portfolio

Corresponding author(s): Christopher E Shaw

Last updated by author(s): 25/10/2022

Reporting Summary

Nature Portfolio wishes to improve the reproducibility of the work that we publish. This form provides structure for consistency and transparency in reporting. For further information on Nature Portfolio policies, see our Editorial Policies and the Editorial Policy Checklist.

Statistics

For all statistical analyses, confirm that the following items are present in the figure legend, table legend, main text, or Methods section.

n/a	Confirmed	
☐	☒	The exact sample size (n) for each experimental group/condition, given as a discrete number and unit of measurement
☐	☒	A statement on whether measurements were taken from distinct samples or whether the same sample was measured repeatedly
☐	☒	The statistical test(s) used AND whether they are one- or two-sided<br>*Only common tests should be described solely by name; describe more complex techniques in the Methods section.*
☐	☒	A description of all covariates tested
☐	☒	A description of any assumptions or corrections, such as tests of normality and adjustment for multiple comparisons
☐	☒	A full description of the statistical parameters including central tendency (e.g. means) or other basic estimates (e.g. regression coefficient) AND variation (e.g. standard deviation) or associated estimates of uncertainty (e.g. confidence intervals)
☐	☒	For null hypothesis testing, the test statistic (e.g. F, t, r) with confidence intervals, effect sizes, degrees of freedom and P value noted<br>*Give P values as exact values whenever suitable.*
☒	☐	For Bayesian analysis, information on the choice of priors and Markov chain Monte Carlo settings
☒	☐	For hierarchical and complex designs, identification of the appropriate level for tests and full reporting of outcomes
☐	☒	Estimates of effect sizes (e.g. Cohen's d, Pearson's r), indicating how they were calculated

Our web collection on statistics for biologists contains articles on many of the points above.

Software and code

Policy information about availability of computer code

Data collection	No software was used
Data analysis	Data analysis was performed in R version 4.0.2 using the packages 'ggplot2' (version 3.3.5) 'rworldmap' (version 1.3-6) and 'survival' (version 3.2-7). Pymol version 1.7.1.1 was used to generate figures of SOD1 dimers.

For manuscripts utilizing custom algorithms or software that are central to the research but not yet described in published literature, software must be made available to editors and reviewers. We strongly encourage code deposition in a community repository (e.g. GitHub). See the Nature Portfolio guidelines for submitting code & software for further information.

Data

Policy information about availability of data

All manuscripts must include a data availability statement. This statement should provide the following information, where applicable:

- Accession codes, unique identifiers, or web links for publicly available datasets
- A description of any restrictions on data availability
- For clinical datasets or third party data, please ensure that the statement adheres to our policy

DATA AVAILABILITY
The raw data used for this study are available under restricted access as per agreement with data contributors and are available on request by contacting alsod@kcl.ac.uk. Summary level data generated by Cox proportional hazards modelling are available in supplementary file 1 and at sod1-alsphen.rosalind.kcl.ac.uk. Source data used to generate figures 2-4 are provided in the Source data file.

CODE AVAILABILITY

The code used for this study is available on request.

Field-specific reporting

Please select the one below that is the best fit for your research. If you are not sure, read the appropriate sections before making your selection.

- [x] Life sciences
- [ ] Behavioural & social sciences
- [ ] Ecological, evolutionary & environmental sciences

For a reference copy of the document with all sections, see nature.com/documents/nr-reporting-summary-flat.pdf

Life sciences study design

All studies must disclose on these points even when the disclosure is negative.

Sample size	We did not perform sample size calculations as we collected all available data globally. For individual variant level analysis we performed regression on all variants with at least 3 cases, reporting those in the main results with 10 cases or more. Effect sizes of variants on the outcomes of interest was not known in most cases in order to perform a power calculation.
Data exclusions	In the case of missing data in covariates or outcomes of interest, those records were excluded from analysis. In the case that we could not determine the genomic location of a variant or the variant was synonymous we excluded that variant from the study.
Replication	Findings were not replicated, for example in an independent cohort, because we have attempted to collect all possible cases globally. For individual variants, the sample size is too small to partition into test/training samples.
Randomization	This was a retrospective observational study assessing whether people with a SOD1 variant have a different survival to people without a SOD1 variant. People cannot be randomized to having a genetic variant or ALS by the researchers so it is not appropriate in this case.
Blinding	Investigators were not blinded to the study groups

Reporting for specific materials, systems and methods

We require information from authors about some types of materials, experimental systems and methods used in many studies. Here, indicate whether each material, system or method listed is relevant to your study. If you are not sure if a list item applies to your research, read the appropriate section before selecting a response.

Materials & experimental systems

n/a	Involved in the study	
x		Antibodies
x		Eukaryotic cell lines
x		Palaeontology and archaeology
x		Animals and other organisms
	x	Human research participants
	x	Clinical data
x		Dual use research of concern

Methods

n/a	Involved in the study	
x		ChIP-seq
x		Flow cytometry
x		MRI-based neuroimaging

Human research participants

Policy information about studies involving human research participants

Population characteristics	People were eligible if they had a recorded diagnosis of ALS made by an ALS specialist, or their diagnosis was published as ALS in the literature.
Recruitment	We primarily accessed the ALS Online Database, a manually curated collection of published evidence about genes and genetic variants associated with ALS (https://alsod.ac.uk).<br>We also contacted clinicians working in specialist centres that performed genetic testing and requested they provide anonymised records of people with SOD1-ALS. Each data source and their local ethical approval are detailed below:<br><br>Macquarie University: participants recruited under informed written consent as approved by the Human Research Ethics Committee of Macquarie University.<br><br>ANZAC Research Institute: participants recruited under informed written consent as approved by the institutional review board of the ANZAC Research Institute (Sydney South West Area Health Service).<br><br>University of Massachusetts: data were acquired with formal patient consent according to protocols reviewed and approved by the Institutional Review Boards of first the Massachusetts General Hospital and then the University of Massachusetts Medical School.<br><br>University Hospitals of Montpellier: all participants consented for storage of their data and its use in research, the study was approved by the Ethics committee (CCPPRB) of Pitié Salpêtrière Hospital n°131/92<br><br>King's College London: participants provided consent for storage of their genetic and clinical data and its use for research in protocols approved by Local Research Ethics Committee approval number 222/02.<br><br>Washington University School of Medicine in St Louis: the data was collected under a waiver of consent since the participants were all deceased.<br><br>Peking University Third Hospital: all patients included provided written informed consent to participate in the clinical and genetic studies, which were approved by the institutional ethics committee of Peking university third hospital (PUTH)<br><br>Northwestern Medicine – Feinberg School of Medicine: Northwestern's Institutional Review Board has reviewed and approved our Neurological Diseases Registry annually since 1991. Consents include the statement that data obtained from studying the subject's contributions may be shared with other researchers as long as the data is deidentified.<br><br>Istituto Auxologico Italiano IRCCS-University of Milan: data were collected in the project SOD1-ITALS approved by Ethical Committee of the IRCCS Istituto Auxologico Italiano<br><br>University of Belgrade: All individuals gave written informed consent for the storage of their data and its use in research and the Ethics Committee of the School of Medicine at the University of Belgrade approved this protocol.<br><br>Koç University: data and sample collection was approved by Boğaziçi University Ethics Committee. Signed informed consent was obtained from all subjects. The storage of the data and its use for research was approved by the patients.<br><br>Project MinE: the Project MinE database was searched for people with ALS in whom SOD1 variants had been identified by whole genome sequencing the ethical approval for the project MinE dataset is described in detail elsewhere.<br>To compare age of onset and survival in people from the general ALS population and SOD1-mediated ALS we used a comparator population of people from population-based datasets of ALS in five European populations (UK, Netherlands, Italy, Ireland and Belgium) and the United States. The data from European countries consisted of clinical variables only that were originally collected and analysed as part of the Survival, Trigger and Risk, Epigenetic, eNvironmental and Genetic Targets for motor neuron Health (STRENGTH) project. The ethical approval for the European and US datasets are described in detail elsewhere.
Ethics oversight	The data analysed in this project were either in the public domain (phenotype information sources from scientific publications) or were fully anonymised at source and therefore completely anonymous at the point of access. No new data were collected for this study. Following King's College London Research Governance protocols ethical clearance was not required for this study.

Note that full information on the approval of the study protocol must also be provided in the manuscript.

Clinical data

Policy information about clinical studies

All manuscripts should comply with the ICMJEguidelines for publication of clinical research and a completedCONSORT checklist must be included with all submissions.

Clinical trial registration	n/a
Study protocol	n/a

Data collection	Data were collected from published research studies or provided to the research team as anonymised clinical datasets.
Outcomes	The outcomes were disease duration and age of onset, these were defined in order to test our study hypotheses.
